# Prenatal maternal infections and children’s socioemotional development: findings from the UK Millennium Cohort Study

**DOI:** 10.1007/s00787-020-01644-y

**Published:** 2020-09-19

**Authors:** Hildigunnur Anna Hall, Lydia Gabriela Speyer, Aja Louise Murray, Bonnie Auyeung

**Affiliations:** 1 Department of Psychology, University of Edinburgh, 7 George Square, Edinburgh, EH8 9JZ UK; 2 Autism Research Centre, Department of Psychiatry, University of Cambridge, Cambridge, CB2 8AH UK

**Keywords:** Pregnancy, Infections, Mothers, Children, Socioemotional development, Mental health

## Abstract

Previous research suggests that prenatal maternal infections may be associated with increased odds of children having a neurodevelopmental disorder. However, little evidence exists on associations with broader child outcomes, especially subclinical symptoms. Participants were the *N* = 14,021 members of the population-representative UK Millennium Cohort Study. We examined associations between prenatal maternal infections, both maternal-reported and hospital-recorded, and children’s socioemotional development, using the Strengths and Difficulties Questionnaire (SDQ) at age three. Maternal-reported prenatal infections were associated with increased emotional symptoms, after adjusting for several potential confounds and covariates. Hospital-recorded prenatal infections were not associated with children’s socioemotional outcomes, after adjusting for potential confounding and covarying factors. Findings suggest that prenatal maternal infections, particularly those which the mothers remember months later, may be associated with increased emotional problems in early childhood. This emphasises the need for screening for and preventing infections during pregnancy. Further, the occurrence of prenatal infection indicates the potential need for early intervention for children’s emotional difficulties.

**Electronic supplementary material:**

The online version of this article (10.1007/s00787-020-01644-y) contains supplementary material, which is available to authorized users.

## Introduction

Although findings remain mixed, previous research has suggested that prenatal maternal infections may be associated with increased odds of children developing neurodevelopmental conditions, such as schizophrenia and autism spectrum disorder (ASD) [for reviews, see 1, 2] and attention-deficit/hyperactivity disorder (ADHD) [3, 4]. However, despite evidence from animal studies indicating that maternal immune activation has potentially broad effects on nervous system development [5], few studies have examined the impact of prenatal infections on other potentially relevant offspring psychological phenotypes.

Numerous studies have assessed links between prenatal maternal infections and neurodevelopmental conditions. Khandaker et al. [2] reviewed studies on links between prenatal infections and the development of schizophrenia, which used both serological assays and clinical examinations to determine exposure to prenatal infections. They concluded that a range of prenatal infections may be associated with increased odds of offspring schizophrenia. Similarly, Jiang et al. [1] reviewed the literature on links between prenatal maternal infections and children’s ASD, with no restrictions on how exposure to infections was assessed. They found a significant overall effect of infections, and a stronger effect for infections requiring hospitalisation, specifically. A few studies have examined links between prenatal infections and ADHD, although the assessment of exposure to infections varied somewhat. Dreier et al. [3] used telephone interviews, where women were asked whether they had experienced any episodes of a range of specific infections or fever during their pregnancy. Their findings showed no overall association between prenatal fever or infections and ADHD. However, they reported significant associations between fever in 9–12 weeks and genitourinary infections in 33–36 weeks, suggesting a timing-specific effect. Ginsberg et al. [6] relied on data from hospital records, where they examined exposure to infections in pregnancy which required inpatient care. They found an overall association between prenatal infections and ADHD; however, this link was fully attenuated in sibling comparisons. Lastly, Mann and McDermott [4] examined clinical billing records to determine prenatal exposure to genitourinary infections specifically, which included infections diagnosed in physicians’ offices as well as hospitals. They reported significant links between prenatal genitourinary infections and children’s ADHD, but did not examine timing-specific associations or carry out sibling comparisons. These methodological differences may perhaps help explain the conflicting findings.

Animal studies suggest that prenatal maternal immune activation can have widespread effects on nervous system development [5], suggesting that in humans it could put offspring at risk of a broader range of developmental issues than those that the majority of previous studies have focused on. A systematic review of population-based studies [7] examined evidence on links between prenatal maternal infections and offspring mood disorders. They found the evidence to be conflicting overall, but argued that discrepancies in findings may, in part, be due to methodological limitations, such as small sample sizes, misclassifications of exposures and unmeasured confounds. The most robust studies showed evidence for a link between prenatal influenza and offspring bipolar disorder [8, 9].

As well as examining exposure to infections in medical records, maternal self-reports and serum samples, recent studies have examined links between exposure to antibiotic medication and child outcomes. A large cohort study [10] found that exposure to antibiotics in pregnancy was associated with a range of offspring psychiatric diagnoses. However, it remains unclear whether these links refer to effects of the antibiotic medication or the infections the drugs are taken for. Similarly, Lydholm et al. [11] examined links between use of antibiotics during pregnancy and offspring mental disorders and reported statistically significant associations.

The most frequently explored child outcomes in relation to prenatal maternal infections are psychiatric diagnoses. However, De Girolamo, Dagani, Purcell, Cocchi and McGorry’s review [12] showed that many mental disorders are not diagnosed until late childhood or even early adulthood and that effective treatment of mental disorders is often not initiated until years after disorder onset. They further argue that early interventions may help reduce symptom severity or persistence of disorders and prevent secondary disorders. Together, these considerations make it important to examine symptoms before they coalesce into clinically diagnosable disorders and/or where they manifest at a subclinical level. Little evidence exists on links between prenatal infections and children’s mental health outcomes outside of clinical diagnoses. Green et al. [13] found a significant association between prenatal infections and increased odds of children’s developmental vulnerabilities at age five across five subdomains: physical, social, emotional, cognitive and communicative after adjusting for other known risk factors.

A limitation of many studies examining associations between prenatal infections and children’s developmental outcomes is that they rely on a single measure of exposure to infections. Frequently used data sources are hospital admission records [e.g. 14, 15] and maternal self-reports [e.g. 16, 17], where the former might be assumed to be restricted to more severe cases than the latter. Previous work [18] has shown that agreement between these two measures in the UK Millennium Cohort Study, data from which we use in the current study, was low (weighted Cohen’s kappa = 0.075). This is in line with studies on other samples [19] and may help explain discrepant findings in the literature.

In the current study, we focus on children’s early signs of a broad spectrum of mental health phenotypes. We examine links between prenatal maternal infections and children’s socioemotional development at age three, measured by the parent-reported Strengths and Difficulties Questionnaire (SDQ) [20], a validated measure of emerging child mental health problems [21–23], in the UK representative Millennium Cohort Study (MCS). Children’s scores on the SDQ, at various ages, have been shown to predict psychiatric diagnoses such as conduct, hyperactivity, depressive and anxiety disorders [22, 23]. In the current study, the pre-school version of the scale was used, which has previously been validated in the MCS [21]. The scale offers an opportunity to identify children’s mental health problems early on, which may in turn lead to early intervention, if required. We draw on data from both hospital admissions and maternal self-reports, to gain a more complete picture of the potential associations between prenatal infections and children’s early indications of mental health problems.

Our aims in the current study were (1) to examine associations between prenatal maternal infections, both hospital-recorded and maternal-reported, and children’s scores on the total difficulties scale of the SDQ at age three and (2) to examine associations between prenatal maternal infections and children’s scores on each of the five subscales of the SDQ: (a) emotional symptoms, (b) conduct problems, (c) hyperactivity/inattention, (d) peer relationship problems and (e) prosocial behaviour. We hypothesised that prenatal infections would be significantly associated with higher total difficulties scores, as well as higher scores on all subscales except prosocial behaviour, where we expected to see associations with lower scores. We further hypothesised that associations between prenatal infections and all child outcomes would be attenuated after adjusting for relevant confounding and covarying factors.

## Method

### Cohort

The UK Millennium Cohort Study (MCS) [24] is a longitudinal birth cohort study which has collected data on over 18,000 children and their families since the start of the twenty-first century, at ages 9 months, 3, 5, 7, 11, 14 and 17 years. Electoral wards in all countries of the UK were used as a basis for sampling and stratification. Disadvantaged families and ethnic minority groups were oversampled to ensure sufficient representation. The data are available to download at https://ukdataservice.ac.uk/. Ethical approvals and informed consent were acquired for all sweeps of data collection [25].

**Fig. 1:**
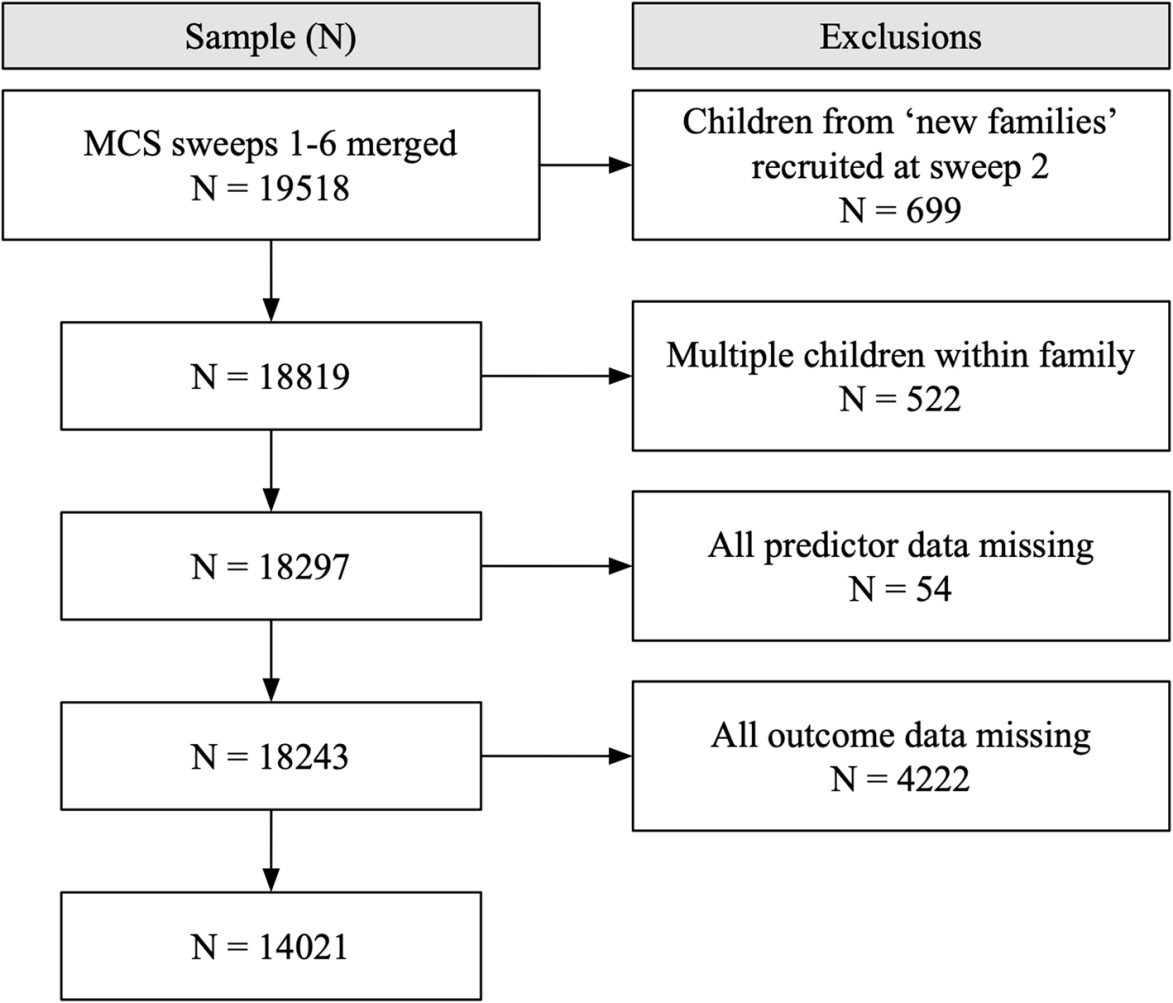
Sample selection procedure

Data from the first two sweeps of the MCS were used in the current study, with a final sample of *N* = 14,021. At the time of the second sweep of the MCS, families of 699 children were recruited and added to the cohort [24]. These ‘new families’ were excluded from the current study as they provided information on key study variables (e.g. prenatal infections) over 2 years later than the original cohort. Families with more than one child taking part in the MCS were also excluded to ensure independence of observations with regard to maternal characteristics and home environment (Fig. 1).

### Measures

#### Maternal-reported prenatal infections

Data on maternal-reported prenatal infections were gathered from interviews which took place at the first sweep of the MCS, when the cohort children were around nine months of age (range 8.1–12.7 months). Mothers were asked, ‘Did you have any illnesses or other problems during your pregnancy that required medical attention or treatment’? If mothers responded positively to this question, they were further asked, ‘What illnesses or problems did you have’? The data made available had already been coded into illness categories, two of which represented infections: (1) Urinary infection and (2) Other/Non-trivial infections [26]. For the purposes of the current study, if either infection code was present, this was coded as ‘infection’. If another illness was identified this was coded as ‘other illnesses’ and negative responses to the initial question of whether the mother had experienced any illness during pregnancy were coded as ‘no illness’.

#### Hospital-recorded prenatal infections

Information on infections diagnosed during hospital admissions in pregnancy was collected from linked maternity hospital records. The data were provided as 3-digit codes using the Information Classification of Diseases, 10th revision (ICD-10) [27]. In the current study, all ICD-10 codes were assessed and those representing infections or inflammatory illness were coded as an indication of hospital-recorded infection (Table S1 in the electronic supplementary material ).

#### Children’s socioemotional outcomes

Children’s socioemotional outcomes were measured using the parent-reported 2- to 4-year-old version of the Strengths and Difficulties Questionnaire (SDQ), a 25-item behavioural questionnaire [20]. The questionnaire consists of five subscales, each containing five items: (1) emotional symptoms (e.g. ‘many worries, often seems worried’), (2) conduct problems (e.g. ‘often has temper tantrums or hot tempers’), (3) hyperactivity/inattention (e.g. ‘restless, overactive, cannot stay still for long’), (4) peer relationship problems (e.g. ‘rather solitary, tends to play alone’) and (5) prosocial behaviour (e.g. ‘considerate of other people's feelings’). A parent of the cohort child, which in the majority of cases (99.9%) was the child’s mother, responded to each of the 25 items using the following options: ‘not true’, ‘somewhat true’, ‘certainly true’, ‘can’t say’ or ‘not applicable’. Total scores for each subscale were computed by summing the item scores and a total difficulties score was computed by summing the total scores from all subscales except the prosocial behaviour scale. Parents responded to the questionnaire at the second sweep of the MCS, when the children were around the age of three (range 32–55 months).

Psychometric properties of the SDQ have been extensively assessed. While the scale has been criticised for limited evidence on cultural validity, criterion validity and test-retest reliability as well as low inter-rater agreement, it has generally shown good discriminative, structural and convergent validity and the total difficulties scale is reported to have good internal consistency [28]. Studies have further shown that the SDQ has good predictive validity, for diagnoses of ADHD [29, 30] and other mental disorders [22, 23, 31]. The pre-school version of the scale, which we use in the current study, has previously been validated in the MCS [21].

#### Confounding and covarying factors

Potential confounders and covariates were selected a priori based on their previously suggested relationships with prenatal maternal infections and/or children’s socioemotional development. Study confounders selected on the basis of their potential relationship with both women’s health during pregnancy and child outcomes were maternal age at birth [32, 33], maternal education and area-based deprivation [34, 35]. Additional study covariates selected on the basis of their potential relationship with children’s socioemotional development were child’s sex [36], child’s age at SDQ assessment, maternal prenatal smoking [37], harsh parenting [38], maternal history of psychiatric illness [39] and maternal postnatal psychological distress [40]. These were included in order to estimate the unique contribution of infection during pregnancy, over and above other factors that may influence child socioemotional outcomes.

Paternal age, education, history of psychiatric illness and postnatal psychological distress were also considered as potential covariates, but were not included in the main models due to low numbers. However, we included sensitivity analyses where these factors were additionally adjusted for. Similarly, we did not adjust for gestational age and birth weight in the main models, as they may act as mediators in the association between prenatal infections and children’s socioemotional outcomes [14, 41, 42]. However, as views may differ on this issue, we additionally adjusted for these factors in sensitivity analyses.

Information on parental age at birth (continuous), parental highest level of completed education (GCSE or equivalent/higher), child’s sex (male/female), maternal prenatal smoking (yes/no) and parental history of psychiatric illness (yes/no) was gathered from interviews from the first sweep of the MCS. Regarding parental history of psychiatric illness, parents were asked ‘Do you have a longstanding illness, disability or infirmity. By longstanding I mean anything that has troubled you over a period of time or that is likely to affect you over a period of time’? If they responded positively to this question, they were asked ‘What is the matter with you’? to which responses were provided as ICD-10 codes. From these codes, we filtered out the mental and behavioural disorders (Chapter F of the ICD-10) to create this (yes/no) variable. Parents who responded negatively to the question of having a longstanding illness, or reported an illness which was not mental or behavioural, were coded as ‘no’. Parental postnatal psychological distress (high/low) was also assessed during the first sweep, using a 9-item version [43] of the Rutter Malaise Inventory [44], where scores of 4 points or more (on a scale from 0 to 9) indicate increased probability of depression or anxiety (labelled here as ‘high’) [45]. Information on deprivation (quintiles) was gathered from Indices of Multiple Deprivation (IMD) calculated from home postcodes at the time of the first sweep of the MCS [46]. Harsh parenting (continuous) was assessed using the parent-reported Straus Conflict Scale [47], where higher scores represent more frequent parental use of harsh conflict tactics.

### Data analysis

Data were restructured and merged to allow for longitudinal analysis (script available at https://github.com/Lydia-G-S/Millennium-Cohort-Study-Data-Restructuring-in-R). Adjustments for clustering (electoral wards), stratification (socioeconomic status and ethnicity), sampling design and attrition bias were performed using the R package *survey* [48]. We further included a finite population correction factor in order to adjust for sampling from a finite population without replacement, in line with MCS guidance [49]. Linear regressions with robust estimators were used to examine whether prenatal maternal infections were associated with children’s scores on the total difficulties scale and the five subscales of the SDQ.

We adopted a hierarchical approach to the analyses: (1) unadjusted models; (2) models adjusted for potential confounders, which may be associated with both women’s health during pregnancy and child outcomes (maternal age, maternal education and deprivation) and (3) models additionally adjusted for potential covariates, which may be associated with children’s socioemotional development (child’s sex, maternal smoking during pregnancy, harsh parenting, maternal history of psychiatric illness and maternal postnatal psychological distress). We fit this third set of models in order to assess the potential effects of prenatal maternal infections on children’s socioemotional outcomes, over and above the other risk factors. Models on maternal-reported infections and hospital-recorded infections were fit separately. We also fit another set of models where a combined infection variable was used as a predictor, representing infections which were either self-reported by the mothers or recorded in hospital. Due to the exploratory nature of this study, we did not correct for multiple comparisons. All data wrangling and analysis were carried out in R [50] and RStudio [51].

## Results

Descriptive statistics for study variables are shown in Tables S2 and S3.

### Maternal-reported infections

Results from unadjusted linear regressions (Model 1) showed that maternal-reported prenatal infections were significantly associated with higher scores on the following scales: total difficulties, conduct problems, emotional symptoms, hyperactivity/inattention, peer relationship problems, but not prosocial behaviour, with which no association was found. These associations held when adjusting for maternal age, maternal education and area-based deprivation (Model 2). When additionally adjusting for child’s sex, age at SDQ assessment, maternal prenatal smoking, harsh parenting, maternal history of psychiatric illness and maternal postnatal psychological distress (Model 3), associations with total difficulties and emotional symptoms remained significant, whereas associations with other subscales did not (Table 1). For full multiple regression results, see Table S4.

**Table 1:** Regression parameters for the effects of maternal-reported prenatal infections on children’s scores on the total difficulties, conduct problems, emotional symptoms, hyperactivity/inattention, peer relationship problems and prosocial behaviour scales

	*b*	*SE*	*p*
Total difficulties			
M1	1.271	0.120	< 0.001
M2	0.934	0.187	<0.001
M3	0.532	0.200	0.008
Conduct problems			
M1	0.399	0.080	< 0.001
M2	0.291	0.072	< 0.001
M3	0.116	0.081	0.154
Emotional symptoms			
M1	0.286	0.063	< 0.001
M2	0.237	0.059	< 0.001
M3	0.180	0.066	0.007
Hyperactivity/inattention			
M1	0.328	0.091	< 0.001
M2	0.219	0.087	0.012
M3	0.077	0.092	0.399
Peer relationship problems			
M1	0.211	0.065	0.001
M2	0.151	0.063	0.017
M3	0.114	0.074	0.123
Prosocial behaviour			
M1	0.009	0.072	0.898
M2	0.010	0.071	0.885
M3	0.031	0.072	0.665

Model 1 (M1) was unadjusted. Model 2 (M2) was adjusted for maternal age, maternal education and area-based deprivation. Model 3 (M3) was additionally adjusted for child's sex, child’s age at SDQ assessment, maternal prenatal smoking, harsh parenting, maternal history of psychiatric illness and maternal postnatal psychological distress

### Hospital-recorded infections

Results from unadjusted linear regressions (Model 1) showed that hospital-recorded prenatal infections were significantly associated with higher scores on the total difficulties, emotional symptoms and peer relationship problems scales. No association was found with scores on the conduct problems, hyperactivity/inattention or prosocial behaviour scales. No associations were significant after adjusting for maternal age, maternal education and area-based deprivation (Model 2) or additionally adjusting for child’s sex, age at SDQ assessment, maternal prenatal smoking, harsh parenting, maternal history of psychiatric illness and maternal postnatal psychological distress (Model 3) (Table 2). For full multiple regression results, see Table S5.

**Table 2:** Regression parameters for the effects of hospital-recorded prenatal infections on children’s scores on the total difficulties, conduct problems, emotional symptoms, hyperactivity/inattention, peer relationship problems and prosocial behaviour scales

	*b*	*SE*	*p*
Total difficulties			
M1	0.682	0.312	0.029
M2	0.530	0.296	0.074
M3	0.235	0.276	0.396
Conduct problems			
M1	0.178	0.120	0.139
M2	0.138	0.111	0.214
M3	0.051	0.106	0.633
Emotional symptoms			
M1	0.226	0.111	0.043
M2	0.202	0.114	0.076
M3	0.172	0.100	0.085
Hyperactivity/inattention			
M1	0.056	0.157	0.720
M2	0.016	0.153	0.917
M3	− 0.134	0.157	0.393
Peer relationship problems			
M1	0.193	0.086	0.025
M2	0.160	0.082	0.053
M3	0.147	0.084	0.081
Prosocial behaviour			
M1	− 0.128	0.110	0.246
M2	− 0.129	0.110	0.243
M3	− 0.025	0.108	0.814

Model 1 (M1) was unadjusted. Model 2 (M2) was adjusted for maternal age, maternal education and area-based deprivation. Model 3 (M3) was additionally adjusted for child's sex, child’s age at SDQ assessment, maternal prenatal smoking, harsh parenting, maternal history of psychiatric illness and maternal postnatal psychological distress

### Maternal-reported or hospital-recorded infections

When a combined infection variable, indicating an infection reported by the mothers themselves or one that was recorded in hospital, was used, unadjusted models (Model 1) showed associations between prenatal infections and higher scores on all subscales except prosocial behaviour, where no association was found. The significant associations held for total difficulties, conduct problems, emotional symptoms and peer problems when adjusting for maternal age, maternal education and area-based deprivation (Model 2). When additionally adjusting for child’s sex, age at SDQ assessment, maternal prenatal smoking, harsh parenting, maternal history of psychiatric illness and maternal postnatal psychological distress (Model 3), associations with total difficulties and emotional symptoms remained significant (Table 3). For full multiple regression results, see Table S6.

**Table 3:** Regression parameters for the effects of hospital-recorded or maternal-reported prenatal infections (combined variable) on children’s scores on the total difficulties, conduct problems, emotional symptoms, hyperactivity/inattention, peer relationship problems and prosocial behaviour scales

	*b*	*SE*	*p*
Total difficulties			
M1	1.060	0.180	< 0.001
M2	0.782	0.159	< 0.001
M3	0.452	0.174	0.010
Conduct problems			
M1	0.327	0.068	< 0.001
M2	0.239	0.062	< 0.001
M3	0.112	0.068	0.100
Emotional symptoms			
M1	0.277	0.056	< 0.001
M2	0.234	0.053	< 0.001
M3	0.181	0.058	0.002
Hyperactivity/inattention			
M1	0.219	0.079	0.006
M2	0.133	0.076	0.080
M3	0.019	0.082	0.815
Peer relationship problems			
M1	0.198	0.054	< 0.001
M2	0.147	0.053	0.006
M3	0.108	0.063	0.084
Prosocial behaviour			
M1	− 0.043	0.062	0.489
M2	− 0.042	0.062	0.499
M3	− 0.007	0.064	0.908

Note. Model 1 (M1) was unadjusted. Model 2 (M2) was adjusted for maternal age, maternal education and area-based deprivation. Model 3 (M3) was additionally adjusted for child's sex, child’s age at SDQ assessment, maternal prenatal smoking, harsh parenting, maternal history of psychiatric illness and maternal postnatal psychological distress

### Sensitivity analyses

When additionally adjusting for paternal age, education, history of psychiatric illness and postnatal distress, in addition to child birth weight and gestational age at birth, associations between maternal-reported prenatal infections and higher scores on the emotional symptoms subscale remained significant (Table S7). No associations were found between hospital-recorded infections and any of the subscale scores in this analysis (Table S8).

## Discussion

This study examined links between prenatal maternal infections, both maternal-reported and hospital-recorded, and children’s socioemotional outcomes at age three, in the population-representative UK Millennium Cohort Study. We found that maternal-reported prenatal infections were significantly associated with more overall socioemotional difficulties (measured with the total difficulties scale), as well as greater conduct, emotional and peer relationship problems and increased hyperactivity/inattention, after adjusting for maternal age, maternal education and deprivation. When additionally adjusting for child’s sex, age at SDQ assessment, maternal smoking during pregnancy, harsh parenting, maternal history of psychiatric illness and maternal postnatal distress, maternal-reported infections remained significantly associated with total difficulties and emotional problems, but not conduct or peer problems or hyperactivity/inattention. This suggests that prenatal maternal infections uniquely contribute to children’s emotional problems but any effect on conduct and peer problems, as well as hyperactivity/inattention, overlaps with other risk factors. No associations were found between maternal-reported prenatal infections and children’s prosocial behaviour. The pattern of results was somewhat different for hospital-recorded prenatal infections. No significant associations were found after adjusting for potential confounding factors or covariates, suggesting that there is no link between prenatal maternal infections recorded in hospital and children’s socioemotional outcomes. Results from models examining associations between a combined infection variable representing infections either maternal-reported or hospital-recorded were similar to the findings from models on maternal-reported infections. Significant associations were found between prenatal infections and total difficulties, conduct problems, emotional symptoms and peer problems after adjusting for potential confounders. However, only the association with total difficulties and emotional symptoms remained significant after additionally adjusting for potential covariates.

Few studies have assessed links between prenatal maternal infections and children’s psychosocial symptoms outside of clinical criteria. Green et al. [13] found significant associations between prenatal maternal infections diagnosed in hospital, and children’s social and emotional vulnerabilities at age five, after adjusting for covariates. Our results contradict these findings as we found no associations between hospital-recorded infections and child outcomes after adjusting for other factors. Our findings support those of Dreier et al. [3] who found no association between maternal-reported prenatal infections at any point during pregnancy and ADHD, after adjusting for similar covarying factors as the current study. The evidence thus suggests that links between prenatal maternal infections and ADHD symptoms are weak to null, regardless of whether symptoms are examined as dimensions or a dichotomous measure of whether or not diagnostic criteria are met. Although few studies have examined links between prenatal infections and early indications of psychosocial difficulties in humans, many animal studies have studied this. In their review, Knuesel et al. [5] concluded that animal models show that prenatal maternal immune activation is a critical risk factor for both neurochemical and wide-ranging behavioural abnormalities in offspring. They further argue that microglial priming may be a key component of this association. Further research is required to resolve these somewhat conflicting findings.

The observed differences in findings from our models on the two measures on exposure to infections suggest that they may measure different things. Hospital-recorded infections can be argued to be more ‘objective’ than self-reports, as they do not rely on patients’ memory or knowledge of their symptoms, as they are clinically assessed and recorded by health professionals. They may also be thought to provide insight into infections associated with severe illness, as they were diagnosed during an admission to hospital. However, the hospital measure in the current study included all infections recorded during the mothers’ stay in hospital and no distinction was made between primary diagnoses and others in the reported models. When separating primary diagnoses from others, we found that of the 331 cases of infections recorded in hospital, only 39 of those were recorded as a primary diagnosis. Therefore, the other 292 infections may not have been the reason for admission and thus we are unable to speculate on how severe they may have been. Of those 39 with a primary diagnosis of infection in hospital, only 6 self-reported an infection during pregnancy. This rhymes with the previously reported [18] low agreement between the two infection measures in the MCS. In the current study, the weighted Cohen’s Kappa = 0.073 between maternal-reported infections and hospital-recorded infections, for those women who had both measures available. This was even lower (0.0073) when only infections captured as primary diagnoses in hospital were included. The mothers may not have been aware of diagnosed infections or remembered them for retrospective reporting nine months after giving birth, especially if the infections were not the reason for hospital admission. The self-report measure offers the opportunity to capture infections that may have affected the women’s daily lives, but not necessarily required an admission to hospital. However, a limitation of this mode of measuring exposure is compounded in its retrospective nature. Lastly, in the case of the MCS, 22% of the sample did not have linked maternity records; thus, this information was missing for a large group. These data may have been missing not at random, which could bias the findings and thus explain the different results from models based on hospital-recorded infections versus those maternal-reported, which had much lower levels of missing data. We are unfortunately unable to draw conclusions about the direction of any such bias as we do not have information on the infection status for those who did not have linked hospital records. Tate et al. [52] analysed consent to the linkage of hospital records in the MCS and found that consent rates were lower among mothers from minority ethnic groups, those with higher degrees or no qualifications and those who were lone parents. In addition, where a translator was needed in the interviews, rates of consent were lower in situations where the translator was male. However, the use of non-response weights in this study should work against these potential biases, as the weights were constructed using variables predictive of missingness.

This study had several other limitations: No multi-informant data were available for children’s scores on the SDQ at age three, so we are, therefore, unable to conclude whether children’s socioemotional outcomes measured in this study would be observed across different settings. Some studies examining associations with conditions, such as ASD and ADHD, have found that the effects of prenatal infections may depend on timing of exposure and type of infectious agent [1, 3]. Others question the emphasis on the prenatal period, showing risk of psychiatric illness in association with maternal infections outside the period of pregnancy [11, 53]. Assessing type and timing of exposure was beyond the scope of our study, as we had no information on the timing of exposure. Further, maternal reports of infection only made a distinction between urinary infections and other infections and we determined that the study would have been underpowered to detect associations with urinary tract infections separately from other infections. Similarly, models on the hospital data were thought to have too little power to detect small associations in subgroup analyses. We did also not assess any potential dose–response effects, that is, whether having a higher number of infections would be associated increased risk of developmental issues. The maternal-reported data did not contain any information of number of infections, and due to low numbers in the hospital data, such analyses would not have had enough power. Furthermore, the study does not take into account women’s use of antibiotics or other treatments for infections, so we are unable to assess how this may have affected observed associations. Lastly, due to low numbers of families with more than one child taking part in the MCS, we were unable to conduct sibling comparisons, which would have been helpful for addressing the effects of family-level confounds [54].

## Conclusion

In conclusion, the current study reports evidence for links between prenatal maternal infections and children’s socioemotional difficulties, in particular emotional and peer relationship problems. Findings suggest that there are some associations between maternal prenatal infections and children’s mental health symptoms which fall outside clinical criteria. Our results further emphasise the need for studies to include more than one measure of exposure to infections. Findings could have implications for health care policy, emphasising the need for prenatal screening for and prevention of infections as well as early identification of children who may be in need of mental health services, but do not receive a clinical diagnosis.

## Electronic supplementary material

Below is the link to the electronic supplementary material.

Supplementary file 1 (PDF 137 kb)

## Data Availability

Data and user guides are publicly available at https://www.ukdataservice.ac.uk.

## Code availability

Code for data wrangling and merging of the data is available at https://github.com/Lydia-G-S/Millennium-Cohort-Study-Data-Restructuring-in-R.
