# Supplementary material for: Prenatal maternal infections and children’s socioemotional development: findings from the UK Millennium Cohort Study
Source: Eur Child Adolesc Psychiatry. 2020 Sep 19;30(10):1641–50. doi: 10.1007/s00787-020-01644-y (PMC8505323; doi:10.1007/s00787-020-01644-y)
Supplement: Supplementary file 1 — (PDF 137 kb) [file 787_2020_1644_MOESM1_ESM.pdf]

Table S1. ICD-10 codes representing infection from prenatal maternal hospital records.

Code	Description
A04	Other bacterial intestinal infections
A49	Bacterial infection of unspecified site
A56	Other sexually transmitted chlamydial diseases
A60	Anogenital herpesviral [herpes simplex] infections
A63	Other predominantly sexually transmitted diseases, not elsewhere classified
A64	Unspecified sexually transmitted disease
B01	Varicella [chickenpox]
B16	Acute hepatitis B
B18	Chronic viral hepatitis
B37	Candidiasis infection
B95	Streptococcus, Staphylococcus, and Enterococcus as the cause of diseases classified elsewher
B96	Other bacterial agents as the cause of diseases classified elsewhere
G00	Meningitis
I38	Emdocarditis
J22	Unspecified acute lower respiratory infection
L08	Other local infections of skin and subcutaneous tissue
N12	Tubulo-interstitial nephritis, not specified as acute or chronic
N30	Cystitis
N73	Other female pelvic inflammatory diseases
N76	Other inflammation of vagina and vulva
N77	Vulvovaginal ulceration and inflammation in diseases classified elsewhere
O23	Infections of genitourinary tract in pregnancy
O86	Other puerperal infections
O91	Infections of breast associated with pregnancy, the puerperium and lactation
R50	Fever of other and unknown origin
T88	Infection following immunization
Z11	Encounter for screening for infectious and parasitic diseases
Z20	Contact with and (suspected) exposure to communicable diseases
Z22	Carrier of infectious disease

Table S2. Frequencies for study variables

	N	% of total N = 14021
Hospital-recorded infections		
Yes	331	2.40%
No	10607	75.70%
Missing	3083	22.00%
Maternal-reported illness		
Infection	1059	7.60%
Other illness	4353	31.00%
No illness	8606	61.40%
Missing	3	0.00%
Hospital-recorded or maternal reported infectio		
Yes	1325	9.50%
No	9859	70.30%
Missing	2844	20.30%
Maternal age at birth (continuous)		
M	28.7	
SD	5.87	
Range	[14–47]	
Missing (N)	1	0.00%
Maternal education		
High (NVQ3 and higher)	6930	49.40%
Low (NVQ2 and lower)	7079	50.50%
Missing	12	0.10%
Paternal age		
M	32.09	
SD	6.13	
Range	[15–68]	
Missing (N)	2191	15.60%
Paternal education		
High (NVQ3 and higher)	5788	41.30%
Low (NVQ2 and lower)	4825	34.40%
Missing	3408	24.30%
Index of Multiple Deprivation (Quintiles)		
Lowest Q	3978	28.40%
20–<40%	3214	22.90%
40–<60%	2471	17.60%
60–<80%	2059	14.70%
Highest Q	2298	16.40%
Missing	1	0.00%

Table S2 (continued).

	N	% of total N = 14021
Child's sex		
Male	7150	51.00%
Female	6870	49.00%
Missing	1	0.00%
Birth weight (grams)		
M	3376.14	
SD	573.46	
Range	[570–6780]	
Missing (N)	6	0.00%
Gestational age (weeks)		
M	39.35	
SD	1.91	
Range	[23.93–42.88]	
Missing (N)	116	0.80%
Child’s age at assessment (months)		
M	37.68	
SD	2.47	
Range	[31.86–54.81]	
Missing (N)	0	0.00%
Maternal smoking during pregnancy		
Yes	3147	22.40%
No	10869	77.50%
Missing	5	0.00%
Harsh parenting (continuous)		
M	19.95	
SD	5	
Range	[7–35]	
Missing (N)	2330	16.60%
Maternal history of psychiatric illness		
Yes	389	2.80%
No	13622	97.20%
Missing	10	0.10%
Maternal postnatal distress		
High	1963	14.00%
Low	11699	83.40%
Missing	359	2.60%
Paternal history of psychiatric illness		
Yes	97	0.60%
No	10375	74.00%
Missing	3549	25.30%
Paternal postnatal distress		
High	914	6.50%
Low	9272	66.10%
Missing	3835	27.40%

Table S3. Mean (M), standard reviation (SD), range and group size (N) for outcome variables by study predictors and covariates.

	Total Difficulties Score				Conduct Problems				Emotional Symptoms			
	M	SD	Range	N	M	SD	Range	N	M	SD	Range	N
Total sample	9.59	5.29	0–32	13622	2.82	2.07	0–10	13966	1.37	1.51	0–10	13940
Hospital-recorded infections												
Yes	10.28	5.48	0–28	323	3.00	2.05	0–9	330	1.57	1.72	0–10	329
No	9.55	5.26	0–32	10314	2.82	2.07	0–10	10566	1.35	1.48	0–10	10556
Maternal-reported infections												
Infection	10.47	5.71	0–30	1032	3.15	2.20	0–10	1055	1.55	1.67	0–9	1055
Other illness	9.72	5.33	0–30	4236	2.83	2.06	0–10	4339	1.38	1.52	0–10	4331
No illness	9.41	5.21	0–32	8351	2.77	2.05	0–10	8569	1.34	1.49	0–10	8551
Hospital- or maternal-reported infection												
Yes	10.40	5.66	0–30	1293	3.11	2.17	0–10	1320	1.55	1.69	0–10	1320
No	9.49	5.22	0–32	9575	2.80	2.06	0–10	9813	1.34	1.46	0–10	9803
Maternal age at birth												
< 25 years	11.62	5.67	0–32	3408	3.56	2.23	0–10	3512	1.69	1.74	0–10	3509
25-35 years	9.07	5.00	0–31	7938	2.61	1.96	0–10	8129	1.30	1.43	0–10	8109
> 35 years	8.42	4.92	0–28	1686	2.44	1.87	0–10	1720	1.18	1.34	0–9	1717
Maternal education												
Low (NVQ2 and lower)	10.78	5.52	0–32	6805	3.18	2.17	0–10	7033	1.59	1.66	0–10	7023
High (NVQ3 and higher)	8.39	4.76	0–30	6805	2.45	1.89	0–10	6921	1.15	1.30	0–9	6905
Paternal age at birth												
< 25 years	11.38	5.52	0–30	1211	3.46	2.22	0–10	1242	1.66	1.71	0–9	1238
25-35 years	9.19	5.05	0–30	6562	2.66	1.95	0–10	6725	1.31	1.47	0–10	6719
> 35 years	8.52	4.88	0–31	3070	2.46	1.90	0–10	3139	1.19	1.36	0–10	3132
Paternal education												
Low (NVQ2 and lower)	10.00	5.31	0–31	4670	2.94	2.07	0–10	4798	1.42	1.57	0–10	4790
High (NVQ3 and higher)	8.38	4.72	0–28	5656	2.42	1.87	0–10	5773	1.19	1.35	0–10	5769
Index of Multiple Deprivation (Quintiles)												
Lowest Quintile	11.24	5.74	0–32	3805	3.31	2.24	0–10	3948	1.70	1.74	0–10	3937
20-<40%	10.07	5.30	0–30	3130	2.97	2.04	0–10	3204	1.44	1.56	0–10	3197
40-<60%	9.00	4.89	0–28	2410	2.66	1.97	0–10	2467	1.24	1.40	0–10	2460
60-<80%	8.53	4.68	0–30	2020	2.44	1.85	0–10	2053	1.18	1.31	0–10	2053
Highest Quintile	7.70	4.42	0–28	2256	2.26	1.85	0–10	2293	1.04	1.14	0–8	2292

	Hyperactivity/inattention				Peer relationship problems				Prosocial behaviour			
	M	SD	Range	N	M	SD	Range	N	M	SD	Range	N
Total sample	3.91	2.36	0–10	13829	1.55	1.59	0–10	13850	7.35	1.89	0–10	13864
Hospital-recorded infections												
Yes	4.03	2.40	0–10	327	1.74	1.61	0–8	326	7.28	1.95	0–10	328
No	3.91	2.36	0–10	10470	1.53	1.58	0–10	10481	7.34	1.88	0–10	10490
Maternal-reported infections												
Infection	4.15	2.42	0–10	1050	1.67	1.67	0–9	1044	7.31	1.92	0–10	1044
Other illness	3.98	2.41	0–10	4291	1.58	1.62	0–10	4305	7.41	1.89	0–10	4315
No illness	3.84	2.33	0–10	8485	1.52	1.57	0–10	8498	7.33	1.88	0–10	8502
Hospital- or maternal-reported infection												
Yes	4.12	2.42	0–10	1314	1.67	1.65	0–9	1307	7.31	1.91	0–10	1308
No	3.90	2.35	0–10	9721	1.52	1.57	0–10	9734	7.34	1.88	0–10	9746
Maternal age at birth												
< 25 years	4.57	2.40	0–10	3469	1.87	1.65	0–10	3492	7.31	1.93	0–10	3486
25-35 years	3.76	2.30	0–10	8051	1.47	1.56	0–10	8057	7.38	1.87	0–10	8070
> 35 years	3.42	2.33	0–10	1707	1.42	1.61	0–10	1705	7.28	1.88	0–10	1708
Maternal education												
Low (NVQ2 and lower)	4.32	2.38	0–10	6931	1.77	1.64	0–10	6973	7.26	1.91	0–10	6979
High (NVQ3 and higher)	3.50	2.27	0–10	6886	1.34	1.51	0–10	6865	7.45	1.86	0–10	6873
Paternal age at birth												
< 25 years	4.47	2.39	0–10	1228	1.87	1.65	0–10	1234	7.36	1.90	0–10	1227
25-35 years	3.82	2.30	0–10	6673	1.47	1.55	0–9	6659	7.39	1.86	0–10	6687
> 35 years	3.51	2.28	0–10	3110	1.40	1.57	0–10	3116	7.33	1.87	0–10	3118
Paternal education												
Low (NVQ2 and lower)	4.08	2.34	0–10	4755	1.62	1.61	0–10	4759	7.32	1.88	0–10	4772
High (NVQ3 and higher)	3.50	2.26	0–10	5734	1.33	1.49	0–10	5721	7.42	1.85	0–10	5730
Index of Multiple Deprivation (Quintiles)												
Lowest Quintile	4.39	2.44	0–10	3878	1.92	1.70	0–10	3920	7.31	1.95	0–10	3903
20-<40%	4.05	2.33	0–10	3178	1.65	1.62	0–9	3177	7.35	1.87	0–10	3185
40-<60%	3.73	2.27	0–10	2443	1.41	1.52	0–10	2441	7.39	1.84	0–10	2454
60-<80%	3.59	2.30	0–10	2045	1.36	1.49	0–10	2037	7.32	1.88	0–10	2035
Highest Quintile	3.36	2.25	0–10	2284	1.10	1.34	0–10	2274	7.43	1.85	0–10	2286

Note. Categorisation of age variables, birth weight and harsh parenting were only used for descriptive purposes, continuous variables were used in regression models. Unweighted descriptives are shown.

Table S3 (continued).

	Total Difficulties Score				Conduct Problems				Emotional Symptoms				Hyperactivity/inattention				Peer relationship problems				Prosocial behaviour			
	M	SD	Range	N	M	SD	Range	N	M	SD	Range	N	M	SD	Range	N	M	SD	Range	N	M	SD	Range	N
Child's sex																								
Male	10.10	5.37	0–32	6933	2.94	2.08	0–10	7121	1.36	1.51	0–10	7111	4.20	2.40	0–10	7054	1.66	1.63	0–10	7048	7.13	1.93	0–10	7057
Female	9.05	5.16	0–30	6688	2.69	2.04	0–10	6844	1.38	1.52	0–10	6828	3.60	2.28	0–10	6774	1.44	1.54	0–10	6801	7.59	1.81	0–10	6806
Birth weight																								
< 2500g	10.93	5.65	0–30	807	3.00	2.12	0–10	848	1.69	1.68	0–10	841	4.50	2.45	0–10	836	1.89	1.79	0–10	836	7.34	1.96	0–10	838
≥ 2500g	9.50	5.26	0–32	12809	2.81	2.06	0–10	13112	1.35	1.50	0–10	13093	3.87	2.35	0–10	12987	1.53	1.57	0–10	13008	7.35	1.88	0–10	13020
Gestational age																								
< 37 weeks	10.26	5.54	0–30	1245	2.89	2.08	0–10	1282	1.52	1.57	0–10	1280	4.23	2.44	0–10	1270	1.70	1.69	0–10	1270	7.27	1.91	0–10	1271
≥ 37 weeks	9.51	5.26	0–32	12266	2.81	2.06	0–10	12571	1.36	1.50	0–10	12546	3.87	2.35	0–10	12446	1.53	1.58	0–10	12465	7.36	1.88	0–10	12479
Child's age at SDQ assessment																								
< 37 months	9.59	5.24	0–31	7428	2.84	2.07	0–10	7607	1.34	1.47	0–10	7582	3.89	2.36	0–10	7531	1.58	1.61	0–10	7562	7.28	1.89	0–10	7552
≥ 37 months	9.58	5.35	0–32	6194	2.79	2.06	0–10	6359	1.41	1.59	0–10	6358	3.94	2.37	0–10	6298	1.51	1.57	0–10	6288	7.44	1.88	0–10	6312
Maternal smoking during pregnancy																								
Yes	11.50	5.63	0–32	3031	3.59	2.24	0–10	3134	1.61	1.65	0–10	3130	4.54	2.43	0–10	3091	1.83	1.66	0–10	3096	7.21	1.95	0–10	3106
No	9.04	5.06	0–30	10586	2.59	1.96	0–10	10827	1.30	1.46	0–10	10805	3.73	2.31	0–10	10733	1.47	1.56	0–10	10749	7.39	1.87	0–10	10753
Harsh parenting																								
High (highest 25%)	11.76	5.33	0–31	2859	3.85	2.08	0–10	2915	1.53	1.61	0–10	2908	4.72	2.42	0–10	2897	1.72	1.63	0–9	2892	6.91	1.87	0–10	2904
Low	8.56	4.86	0–30	8571	2.40	1.87	0–10	8743	1.26	1.40	0–10	8729	3.53	2.22	0–10	8669	1.42	1.53	0–10	8671	7.50	1.84	0–10	8688
Maternal history pf psychiatric illness																								
Yes	11.16	5.70	0–31	372	3.52	2.31	0–10	384	1.51	1.54	0–7	384	4.36	2.56	0–10	379	1.87	1.61	0–8	381	7.20	2.06	0–10	384
No	9.54	5.27	0–32	13240	2.80	2.05	0–10	13572	1.37	1.51	0–10	13546	3.90	2.36	0–10	13440	1.54	1.59	0–10	13459	7.36	1.88	0–10	13470
Maternal postnatal distress																								
High	12.35	5.85	0–31	1877	3.74	2.24	0–10	1955	1.92	1.84	0–10	1946	4.74	2.48	0–10	1925	2.06	1.76	0–10	1923	7.11	1.99	0–10	1930
Low	9.09	5.03	0–32	11404	2.66	1.99	0–10	11655	1.26	1.41	0–10	11638	3.76	2.31	0–10	11558	1.46	1.54	0–10	11573	7.39	1.86	0–10	11584
Paternal history of psychiatric illness																								
Yes	10.32	5.77	1–25	92	3.12	2.19	0–9	95	1.67	1.79	0–8	96	4.06	2.21	0–10	93	1.49	1.50	0–6	96	7.25	1.99	2–10	95
No	9.09	5.05	0–31	10097	2.65	1.98	0–10	10335	1.29	1.45	0–10	10323	3.76	2.31	0–10	10256	1.45	1.55	0–10	10247	7.37	1.86	0–10	10267
Paternal postnatal distress																								
High	10.72	5.72	0–30	879	3.28	2.23	0–10	909	1.62	1.68	0–9	903	4.25	2.43	0–10	901	1.68	1.62	0–9	896	7.25	1.91	0–10	902
Low	8.92	4.95	0–31	9043	2.59	1.94	0–10	9237	1.26	1.42	0–10	9233	3.70	2.30	0–10	9170	1.42	1.53	0–10	9170	7.39	1.85	0–10	9177

Note. Categorisation of age variables, birth weight and harsh parenting were only used for descriptive purposes, continuous variables were used in regression models. Unweighted descriptives are shown.

Table S4. Full multiple regression results for the effects of maternal-reported infections on children's scores on the Total Difficulties, Conduct Problems, Emotional Symptoms, Hyperactivity/Inattention, Peer Relationship Problems, and Prosocial Behaviour scales.

	Total Difficulties			Conduct Problems			Emotional Symptoms			Hyperactivity/Inattention			Peer Problems			Prosocial Behaviour		
Model 1	*b*	*SE*	*p*	*b*	*SE*	*p*	*b*	*SE*	*p*	*b*	*SE*	*p*	*b*	*SE*	*p*	*b*	*SE*	*p*
Infection (Ref. = No illness)																		
Yes	1.271	0.120	<.001	0.399	0.080	<.001	0.286	0.063	<.001	0.328	0.091	<.001	0.211	0.065	.001	0.009	0.072	.898
Other illness (Ref. = No illness)																		
Yes	0.327	0.11869	.007	0.071	0.045	.118	0.031	0.029	.278	0.165	0.052	<.001	0.036	0.037	.329	0.075	0.040	.059
	R^2=0.004			R^2=0.002			R^2=0.002			R^2=0.002			R^2=0.001			R^2<0.001		
Model 2																		
Maternal age	-0.145	0.010	<.001	-0.055	0.004	<.001	-0.019	0.003	<.001	-0.051	0.004	<.001	-0.021	0.003	<.001	-0.007	0.004	.054
Maternal education (Ref. = High)																		
Low	1.439	0.105	<.001	0.410	0.043	<.001	0.257	0.030	<.001	0.538	0.046	<.001	0.265	0.032	<.001	-0.187	0.038	<.001
Deprivation (Ref. = Highest Quintile)																		
Lowest Quintile	2.283	0.186	<.001	0.646	0.072	<.001	0.457	0.053	<.001	0.558	0.085	<.001	0.620	0.053	<.001	-0.115	0.069	.097
20-40%	1.438	0.166	<.001	0.389	0.063	<.001	0.279	0.047	<.001	0.304	0.076	<.001	0.444	0.051	<.001	-0.004	0.063	.951
40-60%	0.807	0.167	<.001	0.231	0.072	.001	0.136	0.043	.002	0.175	0.082	.034	0.249	0.051	<.001	-0.015	0.064	.822
60-80%	0.590	0.145	<.001	0.059	0.064	.354	0.126	0.041	.002	0.133	0.076	.082	0.228	0.044	<.001	-0.079	0.064	.214
Infection (Ref. = No illness)																		
Yes	0.934	0.187	<.001	0.291	0.072	<.001	0.237	0.059	<.001	0.219	0.087	.012	0.151	0.063	.017	0.010	0.071	.885
Other illness (Ref. = No illness)																		
Yes	0.345	0.110	.002	0.078	0.042	.066	0.037	0.028	.193	0.171	0.050	<.001	0.041	0.035	.234	0.072	0.040	.069
	R^2=0.110			R^2=0.075			R^2=0.040			R^2=0.056			R^2=0.049			R^2=0.003		
Model 3																		
Maternal age	-0.088	0.009	<.001	-0.029	0.004	<.001	-0.013	0.003	<.001	-0.029	0.004	<.001	-0.016	0.003	<.001	-0.019	0.004	<.001
Maternal education (Ref. = High)																		
Low	1.320	0.107	<.001	0.394	0.043	<.001	0.214	0.031	<.001	0.503	0.048	<.001	0.238	0.034	<.001	-0.235	0.041	<.001
Deprivation (Ref. = Highest Quintile)																		
Lowest Quintile	1.809	0.178	<.001	0.541	0.069	<.001	0.333	0.050	<.001	0.401	0.087	<.001	0.518	0.056	<.001	-0.121	0.073	.099
20-40%	1.147	0.156	<.001	0.307	0.060	<.001	0.184	0.049	<.001	0.243	0.075	.001	0.390	0.049	<.001	-0.003	0.062	.966
40-60%	0.677	0.150	<.001	0.201	0.066	.003	0.081	0.044	.065	0.146	0.080	.069	0.220	0.051	<.001	0.012	0.066	.858
60-80%	0.414	0.130	.002	-0.001	0.058	.987	0.106	0.042	.011	0.034	0.078	.661	0.236	0.044	<.001	-0.028	0.067	.673
Child's sex (Ref. = Female)																		
Male	0.706	0.095	<.001	0.072	0.035	.039	-0.037	0.031	.223	0.490	0.052	<.001	0.203	0.031	<.001	-0.407	0.038	<.001
Child's age at SDQ assessment	-0.072	0.023	<.001	-0.045	0.009	<.001	0.006	0.007	.416	-0.01	0.011	0.238	-0.024	0.007	<.001	0.052	0.008	<.001
Prenatal smoking (Ref. = No)																		
Yes	1.361	0.137	<.001	0.626	0.056	<.001	0.138	0.040	<.001	0.447	0.067	<.001	0.188	0.042	<.001	-0.116	0.056	.040
Harsh parenting	0.305	0.012	<.001	0.154	0.004	<.001	0.020	0.003	<.001	0.110	0.006	<.001	0.017	0.004	<.001	-0.068	0.004	<.001
Maternal history of psychiatric illness (Ref. = No)																		
Yes	-0.096	0.296	.746	0.101	0.128	.433	-0.097	0.090	.282	-0.173	0.143	.229	0.006	0.108	.957	0.202	0.119	.090
Maternal distress (Ref. = Low)																		
High	2.027	0.168	<.001	0.610	0.068	<.001	0.478	0.054	<.001	0.584	0.079	<.001	0.420	0.058	<.001	-0.206	0.064	.001
Infection (Ref. = No illness)																		
Yes	0.532	0.200	.008	0.116	0.081	.154	0.180	0.066	.007	0.077	0.092	.399	0.114	0.074	.123	0.031	0.072	.665
Other illness (Ref. = No illness)																		
Yes	0.223	0.103	.031	0.006	0.038	.885	0.020	0.029	.480	0.131	0.050	.009	0.047	0.038	.219	0.060	0.040	.139
	R^2=0.233			R^2=0.248			R^2=0.055			R^2=0.134			R^2=0.066			R^2=0.058		

Table S5. Full multiple regression results for the effects of hospital-recorded infections on children's scores on the Total Difficulties, Conduct Problems, Emotional Symptoms, Hyperactivity/Inattention, Peer Relationship Problems, and Prosocial Behaviour scales.

	Total Difficulties			Conduct Problems			Emotional Symptoms			Hyperactivity/Inattention			Peer Problems			Prosocial Behaviour		
Model 1	*b*	*SE*	*p*	*b*	*SE*	*p*	*b*	*SE*	*p*	*b*	*SE*	*p*	*b*	*SE*	*p*	*b*	*SE*	*p*
Infection (Ref. = No)																		
Yes	0.682	0.312	.029	0.178	0.120	.139	0.226	0.111	.043	0.056	0.157	.720	0.193	0.086	.025	-0.128	0.110	.246
	R^2<0.001			R^2<0.001			R^2=0.001			R^2<0.001			R^2<0.001			R^2<0.001		
Model 2																		
Maternal age	-0.139	0.012	<.001	-0.055	0.004	<.001	-0.019	0.003	<.001	-0.048	0.005	<.001	-0.018	0.004	<.001	-0.008	0.004	.057
Maternal education (Ref. = High)																		
Low	1.450	0.115	<.001	0.424	0.048	<.001	0.267	0.032	<.001	0.519	0.050	<.001	0.270	0.036	<.001	-0.180	0.043	<.001
Deprivation (Ref. = Highest Quintile)																		
Lowest Quintile	2.298	0.205	<.001	0.651	0.083	<.001	0.463	0.054	<.001	0.559	0.095	<.001	0.627	0.058	<.001	-0.156	0.072	.030
20-40%	1.346	0.182	<.001	0.368	0.075	<.001	0.245	0.047	<.001	0.281	0.082	<.001	0.444	0.057	<.001	0.015	0.070	.833
40-60%	0.737	0.194	<.001	0.219	0.082	.008	0.111	0.049	.025	0.156	0.096	.104	0.220	0.061	<.001	0.018	0.069	.797
60-80%	0.545	0.171	.002	0.055	0.075	.458	0.140	0.046	.003	0.125	0.089	.164	0.196	0.049	<.001	-0.072	0.068	.285
Infection (Ref. = No)																		
Yes	0.530	0.296	.074	0.138	0.111	.214	0.202	0.114	.076	0.016	0.153	.917	0.160	0.082	.053	-0.129	0.110	.243
	R^2=0.105			R^2=0.074			R^2=0.041			R^2=0.051			R^2=0.048			R^2=0.004		
Model 3																		
Maternal age	-0.082	0.011	<.001	-0.028	0.004	<.001	-0.013	0.003	<.001	-0.026	0.005	<.001	-0.014	0.004	<.001	-0.019	0.004	<.001
Maternal education (Ref. = High)																		
Low	1.332	0.116	<.001	0.396	0.048	<.001	0.230	0.033	<.001	0.481	0.051	<.001	0.257	0.039	<.001	-0.249	0.047	<.001
Deprivation (Ref. = Highest Quintile)																		
Lowest Quintile	1.847	0.203	<.001	0.541	0.079	<.001	0.358	0.054	<.001	0.383	0.098	<.001	0.548	0.063	<.001	-0.166	0.079	.038
20-40%	1.049	0.179	<.001	0.288	0.070	<.001	0.155	0.050	.002	0.201	0.083	.016	0.394	0.057	<.001	0.007	0.071	.919
40-60%	0.634	0.178	<.001	0.196	0.075	.009	0.079	0.050	.118	0.136	0.093	.147	0.186	0.061	.002	0.038	0.073	.606
60-80%	0.394	0.155	.011	0.012	0.067	.855	0.122	0.047	.010	0.035	0.089	.695	0.200	0.049	<.001	-0.034	0.077	.655
Child's sex (Ref. = Female)																		
Male	0.700	0.105	<.001	0.078	0.038	.038	-0.046	0.033	.165	0.488	0.059	<.001	0.186	0.036	<.001	-0.447	0.041	<.001
Child's age at SDQ assessment	-0.066	0.027	.014	-0.044	0.009	<.001	0.005	0.008	.520	-0.005	0.012	.696	-0.026	0.008	.001	0.056	0.008	<.001
Prenatal smoking (Ref. = No)																		
Yes	1.371	0.159	<.001	0.636	0.062	<.001	0.119	0.047	.013	0.461	0.075	<.001	0.185	0.048	<.001	-0.084	0.060	.162
Harsh parenting	0.314	0.013	<.001	0.156	0.004	<.001	0.021	0.004	<.001	0.114	0.006	<.001	0.019	0.004	<.001	-0.068	0.005	<.001
Maternal history of psychiatric illness (Ref. = No)																		
Yes	0.337	0.315	.285	0.248	0.139	.074	-0.047	0.103	.651	0.014	0.149	.925	0.066	0.116	.568	0.142	0.135	.294
Maternal distress (Ref. = Low)																		
High	2.062	0.182	<.001	0.629	0.073	<.001	0.464	0.061	<.001	0.558	0.086	<.001	0.429	0.065	<.001	-0.183	0.071	.010
Infection (Ref. = No)																		
Yes	0.235	0.276	.396	0.051	0.106	.633	0.172	0.100	.085	-0.134	0.157	.393	0.147	0.084	.081	-0.025	0.108	.814
	R^2=0.238			R^2=0.253			R^2=0.056			R^2=0.133			R^2=0.070			R^2=0.063		

Table S6. Full multiple regression results for a sensitivity analysis on the effects of hospital-recorded or maternal-reported infections (combined variable) on children's scores on the Total Difficulties, Conduct Problems, Emotional Symptoms, Hyperactivity/Inattention, Peer Relationship Problems, and Prosocial Behaviour scales.

	Total Difficulties			Conduct Problems			Emotional Symptoms			Hyperactivity/Inattention			Peer Problems			Prosocial Behaviour		
Model 1	*b*	*SE*	*p*	*b*	*SE*	*p*	*b*	*SE*	*p*	*b*	*SE*	*p*	*b*	*SE*	*p*	*b*	*SE*	*p*
Infection (Ref. = No infection)																		
Yes	1.060	0.180	<.001	0.327	0.068	<.001	0.277	0.056	<.001	0.219	0.079	.006	0.198	0.054	<.001	-0.043	0.062	.489
	R^2=0.004			R=0.003			R^2=0.004			R^2=0.001			R^2=0.002			R^2<0.001		
Model 2																		
Maternal age	-0.138	0.011	<.001	-0.054	0.004	<.001	-0.019	0.003	<.001	-0.048	0.005	<.001	-0.018	0.003	<.001	-0.008	0.004	.051
Maternal education (Ref. = High)																		
Low	1.457	0.115	<.001	0.426	0.048	<.001	0.268	0.032	<.001	0.527	0.049	<.001	0.266	0.035	<.001	-0.182	0.042	<.001
Deprivation (Ref. = Highest Quintile)																		
Lowest Quintile	2.290	0.201	<.001	0.638	0.080	<.001	0.468	0.053	<.001	0.561	0.094	<.001	0.626	0.058	<.001	-0.155	0.072	.032
20-40%	1.332	0.179	<.001	0.348	0.073	<.001	0.255	0.048	<.001	0.279	0.081	<.001	0.443	0.056	<.001	0.010	0.069	.889
40-60%	0.751	0.190	<.001	0.212	0.081	.009	0.126	0.047	.008	0.159	0.094	.091	0.223	0.060	<.001	0.021	0.069	.758
60-80%	0.532	0.164	.001	0.036	0.072	.621	0.149	0.045	<.001	0.116	0.086	.176	0.202	0.048	<.001	-0.076	0.068	.262
Infection (Ref. = No infection)																		
Yes	0.782	0.159	<.001	0.239	0.062	<.001	0.234	0.053	<.001	0.133	0.076	.080	0.147	0.053	.006	-0.042	0.062	.499
	R^2=0.108			R^2=0.075			R^2=0.043			R^2=0.052			R^2=0.048			R^2=0.004		
Model 3																		
Maternal age	-0.083	0.011	<.001	-0.029	0.004	<.001	-0.014	0.003	<.001	-0.027	0.005	<.001	-0.014	0.004	<.001	-0.019	0.004	<.001
Maternal education (Ref. = High)																		
Low	1.331	0.116	<.001	0.398	0.047	<.001	0.227	0.033	<.001	0.488	0.051	<.001	0.249	0.039	<.001	-0.250	0.047	<.001
Deprivation (Ref. = Highest Quintile)																		
Lowest Quintile	1.828	0.199	<.001	0.518	0.078	<.001	0.366	0.053	<.001	0.381	0.097	<.001	0.548	0.062	<.001	-0.151	0.079	.055
20-40%	1.040	0.176	<.001	0.266	0.069	<.001	0.170	0.050	<.001	0.200	0.083	.016	0.394	0.056	<.001	0.010	0.069	.882
40-60%	0.642	0.176	<.001	0.188	0.075	.013	0.088	0.048	.067	0.138	0.092	.137	0.192	0.060	.001	0.041	0.073	.574
60-80%	0.369	0.150	.014	-0.008	0.067	.905	0.129	0.045	.004	0.022	0.086	.796	0.200	0.049	<.001	-0.037	0.076	.626
Child's sex (Ref. = Female)																		
Male	0.728	0.105	<.001	0.088	0.038	.021	-0.041	0.033	.224	0.503	0.059	<.001	0.184	0.035	<.001	-0.442	0.040	<.001
Child's age at SDQ assessment	-0.073	0.025	.004	-0.045	0.009	<.001	0.004	0.008	.635	-0.009	0.012	.422	-0.025	0.008	.001	0.054	0.008	<.001
Prenatal smoking (Ref. = No)																		
Yes	1.388	0.161	<.001	0.641	0.061	<.001	0.120	0.048	.013	0.474	0.075	<.001	0.180	0.047	<.001	-0.091	0.059	.122
Harsh parenting	0.310	0.013	<.001	0.154	0.004	<.001	0.020	0.004	<.001	0.112	0.006	<.001	0.019	0.004	<.001	-0.068	0.005	<.001
Maternal history of psychiatric illness (Ref. = No)																		
Yes	0.164	0.300	.584	0.206	0.132	.119	-0.097	0.101	.337	-0.044	0.148	.767	0.052	0.113	.647	0.200	0.131	.127
Maternal distress (Ref. = Low)																		
High	2.104	0.177	<.001	0.640	0.073	<.001	0.484	0.060	<.001	0.554	0.084	<.001	0.443	0.063	<.001	-0.205	0.071	.004
Infection (Ref. = No infection)																		
Yes	0.452	0.174	.010	0.112	0.068	.100	0.181	0.058	.002	0.019	0.082	.815	0.108	0.063	.084	-0.007	0.064	.908
	R^2=0.239			R^2=0.252			R^2=0.060			R^2=0.134			R^2=0.070			R^2=0.062		

Table S7. Full multiple regression results for a sensitivity analysis on the effects of maternal-reported infections on children's scores on the Total Difficulties, Conduct Problems, Emotional Symptoms, Hyperactivity/Inattention, Peer Relationship Problems, and Prosocial Behaviour scales, with additional covariates.

	Total Difficulties			Conduct Problems			Emotional Symptoms			Hyperactivity/Inattentior			Peer Problems			Prosocial Behaviour		
	b	*SE*	*p*	*b*	*SE*	*p*	*b*	*SE*	*p*	*b*	*SE*	*p*	*b*	*SE*	*p*	*b*	*SE*	*p*
Maternal age	-0.047	0.014	.001	-0.011	0.006	.046	-0.007	0.005	.124	-0.015	0.007	.030	-0.010	0.005	.033	-0.027	0.006	<.001
Maternal education (Ref. = High)																		
Low	1.228	0.122	<.001	0.308	0.047	<.001	0.21	0.032	<.001	0.482	0.056	<.001	0.260	0.042	<.001	-0.178	0.049	<.001
Paternal age	-0.024	0.012	.041	-0.012	0.005	.017	-0.004	0.004	.276	-0.008	0.006	.130	-0.002	0.004	.610	-0.005	0.005	.300
Paternal education (Ref. = High)																		
Low	0.408	0.112	<.001	0.161	0.045	<.001	0.008	0.034	.812	0.170	0.055	.002	0.058	0.045	.196	-0.038	0.050	.446
Deprivation (Ref. = Highest Quintile)																		
Lowest Quintile	1.484	0.224	<.001	0.450	0.084	<.001	0.282	0.06	<.001	0.303	0.105	.004	0.456	0.069	<.001	-0.163	0.090	.069
20-40%	1.031	0.174	<.001	0.301	0.070	<.001	0.136	0.053	.010	0.258	0.084	.002	0.330	0.056	<.001	-0.116	0.067	.084
40-60%	0.503	0.168	.003	0.132	0.074	.075	0.078	0.044	.078	0.104	0.086	.226	0.176	0.055	.001	-0.053	0.068	.439
60-80%	0.377	0.145	.010	0.001	0.065	.983	0.108	0.044	.015	0.043	0.082	.598	0.209	0.047	<.001	-0.044	0.073	.544
Child's sex (Ref. = Female)																		
Male	0.723	0.103	<.001	0.081	0.040	.042	-0.04	0.033	.226	0.538	0.056	<.001	0.173	0.035	<.001	-0.374	0.045	<.001
Child's age at SDQ assessment	-0.057	0.029	.049	-0.037	0.010	<.001	0.01	0.008	.211	-0.004	0.013	.746	-0.031	0.008	<.001	0.054	0.009	<.001
Birth weight	<0.001	<0.001	.066	0.000	0.000	.713	<0.001	<0.001	.155	<0.001	<0.001	.004	<0.001	<0.001	.407	<0.001	<0.001	.225
Gestational age	-0.07	0.036	.054	-0.016	0.013	.234	-0.015	0.011	.175	-0.025	0.020	.198	-0.018	0.012	.120	0.035	0.013	.007
Prenatal smoking (Ref. = No)																		
Yes	1.252	0.174	<.001	0.600	0.068	<.001	0.102	0.053	.057	0.420	0.087	<.001	0.170	0.052	.001	-0.084	0.066	.202
Harsh parenting	0.306	0.012	<.001	0.155	0.004	<.001	0.019	0.004	<.001	0.110	0.006	<.001	0.019	0.004	<.001	-0.068	0.005	<.001
Maternal history of psychiatric illness (Ref. = No)																		
Yes	0.125	0.359	.728	0.162	0.149	.277	-0.012	0.117	.918	-0.180	0.179	.316	0.074	0.120	.538	0.183	0.148	.217
Maternal distress (Ref. = Low)																		
High	1.929	0.185	<.001	0.609	0.075	<.001	0.401	0.06	<.001	0.573	0.089	<.001	0.398	0.068	<.001	-0.228	0.072	.002
Paternal history of psychiatric illness (Ref. = No)																		
Yes	-0.4	0.656	.543	-0.070	0.232	.762	-0.066	0.193	.731	-0.180	0.279	.520	-0.213	0.185	.250	-0.020	0.251	.937
Paternal distress (Ref. = Low)																		
High	0.587	0.207	.005	0.290	0.084	<.001	0.201	0.074	.007	0.085	0.099	.392	0.048	0.070	.489	-0.052	0.073	.478
Infection (Ref. = No illness)																		
Yes	0.353	0.211	.095	0.042	0.083	.613	0.18	0.08	.025	0.006	0.102	.956	0.096	0.083	.251	0.007	0.090	.939
Other illness (Ref. = No illness)																		
Yes	0.209	0.112	.063	0.006	0.044	.889	0.018	0.033	.575	0.112	0.053	.037	0.055	0.040	.167	0.042	0.046	.364

Table S8. Full multiple regression results for a sensitivity analysis on the effects of hospital-recorded infections on children's scores on the Total Difficulties, Conduct Problems, Emotional Symptoms, Hyperactivity/Inattention, Peer Relationship Problems, and Prosocial Behaviour scales, with additional covariates.

	Total Difficulties			Conduct Problems			Emotional Symptoms			Hyperactivity/Inattentio			Peer Problems			Prosocial Behaviour		
	b	*SE*	*p*	*b*	*SE*	*p*	*b*	*SE*	*p*	*b*	*SE*	*p*	*b*	*SE*	*p*	*b*	*SE*	*p*
Maternal age	-0.045	0.017	.007	-0.011	0.006	.091	-0.008	0.005	.099	-0.014	0.008	.092	-0.009	0.005	.078	-0.030	0.006	<.001
Maternal education (Ref. = High)																		
Low	1.262	0.132	<.001	0.319	0.053	<.001	0.236	0.035	<.001	0.470	0.058	<.001	0.276	0.048	<.001	-0.209	0.055	<.001
Paternal age	-0.017	0.013	.207	-0.013	0.005	.013	-0.005	0.004	.279	-0.004	0.006	.573	-0.001	0.004	.839	-0.004	0.006	.548
Paternal education (Ref. = High)																		
Low	0.443	0.122	<.001	0.162	0.052	.002	0.011	0.036	.761	0.181	0.060	.003	0.059	0.048	.224	-0.017	0.055	.751
Deprivation (Ref. = Highest Quintile)																		
Lowest Quintile	1.499	0.242	<.001	0.427	0.093	<.001	0.303	0.061	<.001	0.273	0.117	.020	0.502	0.076	<.001	-0.222	0.093	.017
20-40%	0.936	0.198	<.001	0.265	0.081	.001	0.091	0.055	.100	0.258	0.094	.006	0.335	0.063	<.001	-0.103	0.076	.177
40-60%	0.536	0.193	.006	0.130	0.083	.116	0.081	0.050	.108	0.126	0.099	.204	0.170	0.064	.008	-0.025	0.077	.746
60-80%	0.327	0.164	.048	-0.018	0.073	.805	0.120	0.049	.016	0.041	0.090	.648	0.183	0.052	<.001	-0.036	0.084	.667
Child's sex (Ref. = Female)																		
Male	0.828	0.118	<.001	0.081	0.045	.075	-0.022	0.036	.546	0.600	0.066	<.001	0.187	0.040	<.001	-0.431	0.052	<.001
Child's age at SDQ assessment	-0.042	0.032	.192	-0.037	0.010	<.001	0.014	0.010	.148	0.004	0.015	.782	-0.028	0.010	.004	0.053	0.010	<.001
Birth weight	<0.001	<0.001	.016	<0.001	<0.001	.836	<0.001	<0.001	.220	<0.001	<0.001	<.001	<0.001	<0.001	.305	<0.001	<0.001	.320
Gestational age	-0.069	0.038	.068	-0.015	0.014	.295	-0.020	0.011	.082	-0.015	0.022	.493	-0.021	0.013	.118	0.035	0.014	.013
Prenatal smoking (Ref. = No)																		
Yes	1.312	0.200	<.001	0.617	0.072	<.001	0.072	0.058	.217	0.458	0.099	<.001	0.182	0.059	.002	-0.066	0.072	.357
Harsh parenting	0.318	0.014	<.001	0.158	0.005	<.001	0.019	0.004	<.001	0.114	0.007	<.001	0.022	0.005	<.001	-0.070	0.005	<.001
Maternal history of psychiatric illness (Ref. = No)																		
Yes	0.486	0.394	.217	0.269	0.162	.097	0.043	0.133	.744	-0.015	0.190	.938	0.126	0.126	.320	0.096	0.170	.572
Maternal distress (Ref. = Low)																		
High	1.996	0.208	<.001	0.630	0.078	<.001	0.397	0.068	<.001	0.566	0.098	<.001	0.391	0.074	<.001	-0.229	0.080	.004
Paternal history of psychiatric illness (Ref. = No)																		
Yes	0.185	0.718	.796	-0.076	0.235	.747	0.224	0.215	.299	-0.012	0.285	.967	-0.120	0.230	.603	-0.038	0.276	.892
Paternal distress (Ref. = Low)																		
High	0.461	0.242	.057	0.285	0.092	.002	0.127	0.081	.118	0.026	0.108	.808	0.049	0.073	.501	-0.018	0.085	.833
Infection (Ref. = No)																		
Yes	0.277	0.327	.397	0.136	0.124	.275	0.179	0.116	.122	-0.222	0.177	.210	0.189	0.102	.065	-0.124	0.130	.341
